# Strategic insights into organ donation: perceptions, attitudes, and the impact of disincentive removal in current and future medical professionals

**DOI:** 10.3389/fpubh.2025.1691544

**Published:** 2025-11-18

**Authors:** Marco A. Arizmendi-Villarreal, Ana C. Ugalde-Flores, Javier Sanchez-Maldonado, Allina P. Flores-Mendoza, Homero A. Zapata-Chavira, Gerardo E. Muñoz-Maldonado, Edelmiro Perez-Rodriguez, Francisco J. Reyna-Sepulveda

**Affiliations:** 1 Department of General Surgery, Hospital Universitario “Dr. José Eleuterio González”, Universidad Autónoma de Nuevo León, Monterrey, Mexico; 2 Department of Transplantation, Hospital Universitario “Dr. José Eleuterio González”, Universidad Autónoma de Nuevo León, Monterrey, Mexico

**Keywords:** organ transplantation, tissue and organ procurement, living donors, health knowledge, attitudes, practice, health care disparities

## Abstract

**Introduction:**

Organ transplantation is a life-saving intervention for patients with end-stage organ failure, yet donation rates remain critically low in many countries. A better understanding of the medical community’s perceptions and the barriers to donation, especially disincentives for donation, is crucial for improving organ procurement.

**Methods:**

We conducted a cross-sectional, observational study using a 22-item structured survey to assess knowledge, attitudes, and disincentives to organ and tissue donation among 686 participants, including medical students, residents, and healthcare personnel from the Universidad Autónoma de Nuevo León.

**Results:**

Most of the participants (81.6%) were willing to donate organs posthumously, and 72.3% expressed willingness to donate during life. However, 54.4% were unaware that the family had the final decision regarding donation. Common disincentives for living donation included concerns about long-term health impacts (23.9%), risk of chronic kidney disease (21%), and fear of death during nephrectomy (19.5%). Notably, 92% believed living donors should receive social or economic support, without perceiving this as conflicting with altruism. Male sex (OR = 2.06; 95% CI: 1.36–3.10) and lack of prior education on donation (OR = 2.54; 95% CI: 1.68–3.84) were significantly associated with unwillingness to donate (*p* ≤ 0.001).

**Discussion:**

Although attitudes toward organ donation are generally favorable among future healthcare professionals, significant knowledge gaps and systemic barriers persist. Implementing structured educational programs, trust-building measures, and policies that remove financial disincentives could strengthen Mexico’s culture of donation and improve transplant outcomes.

## Introduction

Organ transplantation is a life-saving intervention for individuals with end-stage organ failure, yet organ donation rates remain Critical (1, 2). Despite ongoing efforts to increase organ donations through public awareness campaigns and policy reforms, the gap between organ supply and demand persists. In the U.S., approximately 13 patients die every day while waiting for a transplant, a number that could be higher in Mexico, underscoring the urgent need for strategies to increase donations (3–6).

In Mexico, however, beyond public awareness and infrastructure challenges, the legal framework itself shapes donation outcomes. Under the Reglamento de *la Ley General de Salud en Materia de Trasplantes* (Title Second, Chapter I, Article 10), organ recovery from deceased donors requires family consent even when the individual had previously expressed willingness to donate. Thus, although donor intent is recognized, in practice the family has the final decision, which can limit the number of effective donations (7). This highlights the need for healthcare professionals who not only understand the donation process but can also effectively communicate with families to support informed decisions at the time of donation.

Healthcare professionals play a crucial role in facilitating organ donation, as their knowledge and attitudes directly influence public perceptions and the success of donation initiatives. Positive attitudes and accurate information among medical professionals can help improve donor identification, patient counseling, and public trust. However, studies have shown that many healthcare providers have limited knowledge of organ donation processes, which can hinder their ability to promote donation effectively (8–12).

Living kidney donation is challenging due to various disincentives, such as concerns over long-term health consequences and financial burdens. These factors, along with lack of knowledge, deter many potential donors. Providing support for living kidney donors, such as financial reimbursement or health insurance coverage, could help mitigate these barriers and encourage donation (13). This study examines the knowledge, attitudes, and perceived barriers to organ donation among current and future health professionals in Mexico, with a focus on living kidney donation and potential support mechanisms.

## Materials and methods

This was a cross-sectional, observational, and descriptive study conducted between October and December 2023. Participants included undergraduate medical students, residents, physicians, nurses, administrative staff, and allied health personnel from the Faculty of Medicine and University Hospital of the Universidad Autónoma de Nuevo León. A total of 686 participants were recruited through non-probabilistic convenience sampling. Informed consent was obtained electronically. Ethical approval was granted by the institutional review board (Approval No. TR23-00002).

A 22-item, multiple-choice questionnaire was developed based on a literature review and refined through expert consultation with the institutional transplant committee. A pilot study involving 50 participants was conducted to ensure clarity and relevance. The survey comprised four domains: sociodemographic characteristics, knowledge of organ donation, attitudes toward donation, and disincentives for living kidney donation. It was distributed online via institutional platforms and social media (Facebook, Instagram, WhatsApp). Incomplete responses were excluded.

All data was anonymized and securely stored. No identifiable personal information was collected. Access to raw data was restricted to the research team. The study complied with local and international data protection regulations, including GDPR principles where applicable.

Descriptive statistics were used to report frequencies, percentages, and means with standard deviations. The Kolmogorov–Smirnov test was used to assess normality. Group comparisons were conducted using Pearson’s *χ*^2^ 2 × 2 tables test for categorical variables. A *p*-value ≤0.05 was considered statistically significant. Microsoft Excel, Version 2024 (Microsoft Corp., Redmond, WA) and IBM SPSS, Version 27 (IBM Corp., Armonk, NY), were used for data organization, management, and conducting statistical analysis.

A binary logistic regression was conducted to identify the effect associated with sociodemographic and educational predictors of willingness to donate as the dependent variable. Independent variables included age group, sex, religion, occupation, and whether participants had received previous classes or talks on organ and tissue donation. The model was adjusted using the age 18–24 group, female, Catholic, undergraduate students, and previous education on organ donation as reference categories, as these corresponded to the groups with the highest frequency. All variables were entered simultaneously using the *enter* method. Model fit was evaluated through the Hosmer–Lemeshow goodness of fit test and pseudo *R*^2^ measures (Cox & Snell and Nagelkerke). Odds ratios [Exp(B)] with 95% confidence intervals were calculated to estimate the strength and direction of associations. Ninety-five percent confidence intervals and *p*-values were calculated for each predictor. A *p*-value ≤0.05 was considered statistically significant. The analysis was performed using IBM SPSS, Version 27 (IBM Corp., Armonk, NY).

Approval was obtained from the institutional review board and ethics committee of the Universidad Autónoma de Nuevo León (Approval No. TR23-00002). Also, this study complied with the Belmont Report, the Helsinki Declaration of 1964 and Nuremberg Code. All participants provided informed consent before enrollment in the study through the digital survey. The Strengthening the Reporting of Observational studies in Epidemiology guidelines was used to conduct this study (14).

## Results

A total of 686 participants were included in the study. The sex distribution was 428 females (62.4%) and 258 males (37.6%), with a mean age of 22.6 ± 5.1 years. Most participants were medical students 600 (87.5%), followed by residents 62 (9.0%) and healthcare staff 24 (3.5%). Based on religious belief, 457 participants identified as Catholic (66.6%), while 69 identified as Christian (10.1%), 138 reported no religious affiliation (20.1%) and 22 reported “other” religion (3.2%).

A total of 402 participants (58.6%) had received prior education about organ donation. Only 263 respondents (38.3%) correctly identified the National Transplant Center (CENATRA) as the organization responsible for organ donation in Mexico. Concerning the cause of death, 456 respondents (66.5%) considered organ donation feasible following cardiac arrest, whereas 619 (90.2%) recognized brain death as an irreversible condition. Additionally, 375 participants (54.7%) were unaware that the final decision regarding organ donation rests with the family, even when the potential donor had expressed their wishes even in a legal document (see Table 1).

**Table 1:** Knowledge about organ donation.

Questions	*n* (%)
Have you received classes or talks about organ and tissue donation?	
Yes	402 (58.6)
No	284 (41.4)
Do you know which organization is responsible for organ donation in Mexico?	
Yes	263 (38.3)
No	423 (61.7)
Is it possible for someone diagnosed as brain dead to recover?	
Yes	67 (9.8)
No	619 (90.2)
Is it possible to donate organs when the cause of death is cardiopulmonary arrest?	
Yes	456 (66.5)
No	230 (33.5)
The relatives have the last word in deciding on the donation despite the existence of a document expressing the will to donate.	
Yes	311 (45.3)
No	375 (54.7)

Attitudes toward organ donation are summarized in Table 2. A total of 560 participants (81.6%) expressed willingness to become organ donors posthumously, while 496 (72.3%) were open to donating during their lifetime. Among those considering living donation, blood donation was the most frequent (*n* = 469; 30%), followed by bone marrow (*n* = 324; 20.8%) and kidney donation (*n* = 325; 20.8%) were the most acceptable forms of living donation. Despite their willingness, 126 participants (18.4%) expressed rejection to becoming a donor. The primary reasons for indecision included a lack of trust in the donation system (*n* = 55; 19.7%), insufficient knowledge about the donation process (*n* = 44; 15.8%), and concerns about not receiving adequate medical care in an emergency due to their donor status (*n* = 34; 12.2%).

**Table 2:** Attitudes towards organ donation.

Questions	*n* (%)
Would I like to become an organ and tissue donor?	
Yes	560 (81.6)
No	126 (18.4)
If the answer to the previous question is *NO*, for what reason would you not donate? (select as many as you consider necessary)	
Lack of confidence in the organ donation and transplantation system	55 (19.7)
Lack of knowledge about donation	44 (15.8)
If I am a donor, I will not receive medical care in case of an emergency	34 (12.2)
Fear of organ removal while alive	32 (11.5)
Concern about human organ trafficking	32 (11.5)
Concern about family disapproval	26 (9.3)
Physical mutilation upon death	24 (8.6)
Not specified	13 (4.7)
Illness	10 (3.6)
Religious belief	9 (3.2)
Do you have a document expressing your willingness to donate?	
Yes	141(20.6)
No	545(79.4)
If the answer to the previous question is *YES*, which document do you have?	
Driver’s license	112 (54.1)
Verbal expression	43 (20.8)
CENATRA’S Official format	26 (12.6)
Donor card	26 (12.6)
Have you talked to your family about this decision?	
Yes	393 (57.3)
No	293 (42.7)
If it were your decision, would you authorize the organ donation of a family member without knowledge of his or her prior wishes?	
Yes	373 (54.4)
No	313 (45.6)
Would you donate an organ or tissue in life?	
Yes	496 (72.3)
No	190 (27.7)
Which one would you donate in life?	
Blood	469 (30.0)
Kidney	325 (20.8)
Bone marrow	324 (20.8)
Liver	307 (19.7)
Lung	130 (8.3)
Not specified	6 (0.4)

Based on multiple-response answers, the main disincentives and support strategies for living kidney donors are presented in Table 3. The three most frequent disincentives for living kidney donation are: a potential decline in long-term quality of life (*n* = 492; 23.9%), the risk of developing chronic kidney disease (*n* = 432; 21.0%), and fear of death during nephrectomy (*n* = 402; 19.5%). Furthermore, 631 participants (92.0%) expressed that living kidney donors should receive social or economic support, which they did not perceive as conflicting with the altruistic nature of organ donation. The most frequently preferred forms of support included access to medical services (*n* = 591; 28.7%), coverage of surgery and hospitalization expenses (*n* = 573; 27.8%), and priority placement on the transplant waiting list in cases of developing chronic kidney disease (*n* = 452; 22.0%).

**Table 3:** Perceived disincentives to living kidney donation and opinions on support strategies among Mexican respondents.

Questions	*n* (%)
Select the three disincentives (barriers) to living kidney donation that you consider most important.	
Long-term decrease in life quality	492 (23.9)
Risk of chronic kidney disease	432 (21.0)
Risk of dying during kidney removal	402 (19.5)
Concern that the donor, a family member or friend will need a kidney in the future	298 (14.5)
High cost for travel and lodging near a transplant center	153 (7.4)
Loss of income during postoperative recovery	142 (6.9)
Pain and discomfort due to surgery	139 (6.8)
Do you think that social and economic support should be offered to people who donate a kidney during their lifetime?	
Yes	631 (92.0)
No	55 (8.0)
What kind of support do you think should be offered to living kidney donors? (select the 3 that you think are most important)	
Medical service for surveillance studies and complications following kidney donation	591 (28.7)
Support for donor surgery and hospitalization expenses	573 (27.8)
In case of renal insufficiency, have priority on the waiting list	452 (22.0)
Salary compensation and extended disability after donor surgery	326 (15.8)
One-year life insurance	116 (5.6)
By giving these supports against disincentives, do you consider that it goes against the principle of altruism in donation?	
Yes	94 (13.7)
No	592 (86.3)

Participants with prior education on donation were significantly more likely to: know CENATRA’s role (50.7% vs. 20.8%; *p* ≤ 0.001) and who has the final decision to donate despite the existence of a document (53.0% vs. 34.5%; *p* ≤ 0.001). Similarly, a higher prevalence of documents expressing the willingness to donate was reported among the informed participants (26.4% vs. 12.3%; *p* ≤ 0.001), and a higher frequency of conversations about this decision with family members (64.2% vs. 47.5%; *p* ≤ 0.001) (see Table 4).

**Table 4:** Association between having received education on organ and tissue donation and respondents’ knowledge and willingness to donate.

Questions	Yes—received education (*n* = 402)	No—did not receive education (*n* = 284)	*p* value
Do you know which organization is responsible for organ donation in Mexico?			<0.001
Yes	204 (50.7%)	59 (20.8%)	
The relatives have the last word in deciding on the donation despite the existence of a document expressing the will to donate.			<0.001
Yes	213 (53.0%)	98 (34.5%)	
Would I like to become an organ and tissue donor?			<0.001
Yes	352 (87.6%)	208 (73.2%)	
Do you have a document expressing your willingness to donate?			<0.001
Yes	106 (26.4%)	35 (12.3%)	
Have you talked to your family about this decision?			<0.001
Yes	258 (64.2%)	135 (47.5%)	

Education refers to having received talks or classes about organ and tissue donation. Only affirmative (“Yes”) responses are shown. *p* values from chi-squared test.

The chi-square test performed to assess the relationship between religious affiliation and willingness to become a donor was no statistically significant association between the two variables, *χ*^2^(3, *n* = 686) = 4.71, *p* = 0.195.

The logistic regression model was statistically significant [*χ*^2^(9) = 43.09, *p* ≤ 0.001]. The model explained approximately 6.1% (Cox & Snell *R*^2^) to 9.9% (Nagelkerke *R*^2^) of the variance in willingness to donate and demonstrated an adequate fit to the data [Hosmer–Lemeshow *χ*^2^(7) = 3.68, *p* = 0.816]. Males were 2.06 times more likely to express unwillingness to donate compared with females (OR = 2.06; 95% CI: 1.37–3.10; *p* ≤ 0.001). Those not exposed to organ donation classes or talks were 2.54 times more likely to express unwillingness to donate compared to those who were exposed (OR = 2.54; 95% CI: 1.68–3.84; *p* ≤ 0.001). No statistically significant associations were observed for age group (*p* = 0.237), religion (*p* = 0.159), or occupation (*p* = 0.946) (see Table 5).

**Table 5:** Binary logistic regression of independent predictors of willingness to become a donor.

Predictor variable (*n* = 686)	Reference category	*B*	Adjusted OR (95% CI)	*p* value
Age				0.237
25–44	18–24	−0.294	0.74 (0.38–1.45)	0.388
45–63		1.281	3.60 (0.48–26.79)	0.211
Sex				
Male	Female	0.723	2.06 (1.36–3.10)	<0.001
Religion				0.159
Christian	Catholic	0.388	1.47 (0.79–2.72)	0.217
Other		−0.274	0.76 (0.24–2.40)	0.641
None		−0.458	0.63 (0.36–1.10)	0.106
Role				0.946
Graduate	Undergraduate	–	–	–
Health worker		−0.159	0.85 (0.33–2.17)	0.738
		−0.067	0.93 (0.25–3.42)	0.919
Previous education on organ and tissue donation				
No	Yes	0.932	2.54 (1.68–3.84)	<0.001

## Discussion

Our study found that most participants exhibited positive attitudes toward organ donation, with a strong willingness to donate organs posthumously and a significant number to living donation. However, knowledge gaps were prevalent, particularly regarding the legal aspects of organ donation, including the fact that the final decision remains in the hands of the family even when the deceased had signed a legal document expressing consent, which reflects the influence of family veto power in Mexico. These gaps suggest that educational strategies within academic and clinic settings have the potential to improve understanding and promote positive donation attitudes, highlighting the need for structured interventions at both undergraduate and postgraduate levels (15–17). This is particularly relevant in the context of medical education, where a lack of in-depth training and culture on organ donation and transplantation processes could lead to missed opportunities for healthcare professionals to educate patients and their families properly (18).

A major concern identified in this study was the presence of financial and logistical barriers to living kidney donation. Many participants expressed concerns about the long-term health impacts, risk of dying during surgery, and cost of surgery and recovery. This is consistent with findings from other studies, which show that financial burdens significantly deter potential donors (19). Nevertheless, there was strong support for providing non-financial remuneration, including covering surgery-related expenses and offering medical follow-up for donors. This aligns with international recommendations that call for eliminating financial disincentives and offering support to living donors without compromising the altruistic nature of donation (20). Moreover, 92% of participants supported social and economic aid for living donors, particularly access to medical service following donation, economic support for donor surgery and hospitalization, and priority on the waiting list, which are the most accepted support that the participants think should be offered to them, highlighting the perceived fairness in supporting those who help others in need.

Although the participants were healthcare professionals or students, nearly one in ten (9.8%) still perceived brain death as reversible, a misconception that can contribute to confusion, fear and hesitancy, remaining a significant barrier (11, 21). This finding reflects the need to strengthen education on brain death determination and communication, which fosters misconceptions and myths surrounding it, ultimately undermining trust in the organ donation process. Broader donation education for students remains essential to increase donation rates. Strengthening education and access to accurate information could help mitigate these misconceptions, increase transparency in the organ allocation process, build public confidence in the transplantation system, and increase individuals’ willingness to donate.

In our study, the primary reasons for rejection toward organ donation included a lack of trust in the donation system, insufficient knowledge about the donation process, and fears of receiving substandard emergency care due to being identified as a donor. Even though 81.6% of the population would like to be organ donors, only 20.6% have expressed their desire to become one through a legal document. This study reflects an unfriendly system in which, despite the population’s acceptance of donation, few will actually become organ donors. The fragmentation of Mexico’s healthcare system, contributes to bad experiences, presence of myths, and the lack of opportunities for access to essential medical services, such as organ donation and transplantation, particularly within the public sector, which faces inadequate infrastructure, limited implementation of effective public awareness, and restrictive public policies (22–26). These findings also highlight how healthcare fragmentation may undermine efficiency and public transparency in the donation system, emphasizing the need for eliminate it cultural, legal and procedural clarity to reduce public mistrust and family donation conflicts.

Therefore, our study suggests that structural reforms in healthcare and transplant policies could be crucial in addressing some of the barriers to living kidney donation. While the financial support for donors is a significant aspect, it is also important to consider policies that reduce the administrative burden for potential donors, such as streamlining the process for medical leave or insurance coverage (27, 28). The provision of clear, accessible information about the donation process, including donor protections and rights, is another key area where reform is needed (27). Several countries have successfully implemented programs that provide extensive donor support, such as health insurance coverage for donors or guaranteed paid leave (29–32).

The logistic regression identified two key factors associated with the willingness to become a donor among current and future health professionals: female sex, consistent with previous evidence indicating stronger prosocial tendencies in the context of organ donation (33). Second, the transformative potential of formal education to shape positive attitudes toward donation. In contrast, age, religion and occupation were not significantly associated with willingness to donate. Given the homogeneous nature of the sample, the absence of significant associations may reflect the young adults who share similar biomedical and ethical education. This may reflect the population characteristics rather than a true absence of effect on.

Although programs promoting organ donation and transplantation awareness exist within Mexican healthcare institutions, they remain sporadic and often lack a strategic focus. Comprehensive policy reforms are therefore essential. Such reforms should encompass financial support mechanisms for donors and their families, sustained public education and awareness campaigns, investment in hospital infrastructure, targeted training for healthcare professionals, and the strengthening of organ procurement systems. Additionally, integrating organ donation and transplantation topics into the curricula for technical staff, nurses, primary care physicians, and specialists is crucial to ensure a well-prepared and committed healthcare workforce. These measures could significantly increase donation rates in Mexico and help bridge the gap between organ supply and demand. By addressing both the individual and societal factors that influence donation decisions, Mexico can create a more supportive environment for organ donation, making it easier for healthcare professionals, families, and potential donors to make informed decisions that could save lives.

This primary study highlights the need for improved education and policy reforms to increase organ donation rates, particularly living kidney donation and deceased donors. Whereas the surveyed healthcare professionals show positive attitudes toward donation, significant knowledge gaps exist, especially regarding legal aspects and donor rights. Financial, structural and logistical barriers, including concerns about lost wages and surgery-related costs, deter potential living donors. Addressing these barriers through educational and cultural programs, financial support for donors, and fostering family involvement in decision-making could significantly enhance organ donation rates in Mexico, helping to bridge the gap between organ supply and demand.

## Data Availability

The raw data supporting the conclusions of this article will be made available by the authors, without undue reservation.
